# Screening of variants for lactase persistence/non-persistence in populations from South Africa and Ghana

**DOI:** 10.1186/1471-2156-10-31

**Published:** 2009-07-05

**Authors:** Suvi Torniainen, M Iqbal Parker, Ville Holmberg, Elisa Lahtela, Collet Dandara, Irma Jarvela

**Affiliations:** 1 Department of Medical Genetics, University of Helsinki, Helsinki, Finland; 2 International Centre for Genetic Engineering and Biotechnology and Division of Medical Biochemistry, University of Cape Town, Anzio Road, Observatory, 7925, Cape Town, South Africa; 3 Department of Bacteriology and Immunology, University of Helsinki, Helsinki, Finland; 4 Helsinki University Hospital, Laboratory Services, Helsinki, Finland

## Abstract

**Background:**

Lactase non-persistence is a condition where lactase activity is decreased in the intestinal wall after weaning. In European derived populations a single nucleotide polymorphism (SNP) C/T_-13910 _residing 13.9 kb upstream from the lactase gene has been shown to define lactase activity, and several other single nucleotide polymorphisms (G/C_-14010 _T/G_-13915_, C/G_-13907 _and T/C_-13913_) in the same region have been identified in African and Middle East populations.

**Results:**

The T_-13910 _allele most common in European populations was present in 21.8% mixed ancestry (N = 62) individuals and it was absent in the Xhosa (N = 109) and Ghana (N = 196) subjects. Five other substitutions were also found in the region covering the previously reported variants in African and Middle East populations. These included the G/C_-14010 _variant common in Kenyan and Tanzanian populations, which was present in 12.8% of Xhosa population and in 8.1% of mixed ancestry subjects. Two novel substitutions (C/T_-14091 _and A/C_-14176_) and one previously reported substitution G/A_-13937 _(rs4988234) were less common and present only in the Xhosa population. One novel substitution G/A_-14107 _was present in the Xhosa and Ghanaian populations. None of the other previously reported variants were identified.

**Conclusion:**

Identification of the G/C_-14010 _variant in the Xhosa population, further confirms their genetic relatedness to other nomadic populations members that belong to the Bantu linguistic group in Tanzania and Kenya. Further studies are needed to confirm the possible relationship of the novel substitutions to the lactase persistence trait.

## Background

Lactase non-persistence is a condition where the activity of lactase declines during childhood leading to malabsorption of lactose [1]. Certain DNA-variants, however, enable individuals to never lose their lactase activity. A single nucleotide polymorphism (SNP) C/T_-13910 _residing in intron 13 of the MCM6 gene 13.9 kb upstream from the lactase gene (LCT) has been found to correlate perfectly with lactase activity [2-5]. Functional studies have shown that the C/T_-13910 _variant acts as an enhancer [6,7] and it regulates the LCT gene at the transcriptional level [3,8]. This long distance regulatory region seems to be a binding site for the Oct-1 transcription factor [9]. Recently, other variants close to the original variant C/T_-13910 _in the same enhancer region have been identified in nomadic pastoralist and non-pastoralist groups from East Africa, Cameroon and Middle East populations [10-12]. The G/C_-14010 _variant is common in East Africa. The indirect lactose tolerance test (LTT) has been used to show that this variant has a significant association with the lactase persistence trait. Other variants associated with lactase persistence in Africa or the Middle East are T/G_-13915_, C/G_-13907 _and T/C_-13913. _Functional in vitro studies using dual-luciferase reporter assays have shown that the mutant C_-14010_, G_-13907 _and G_-13915 _alleles increase LCT promoter expression [10]. Tissue samples from intestinal biopsies on individuals from the Arabian Peninsula have been used to show a correlation between allele G_-13915 _and lactase activity [11].

To further investigate genetic variants in African populations we sequenced the 400 bp region covering the previously identified variants from DNA-samples of subjects from Xhosa or mixed ancestry groups from South Africa. As a comparison, we sequenced the same region in the Akan people, a Western African linguistic group, which has a long history of farming and trading, but no dairy culture.

## Results

**Figure 1:**
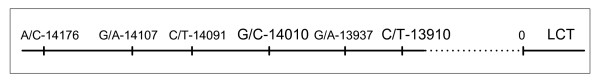
**Locations of single nucleotide substitutions found in the study**. Variants previously associated with lactase activity are shown in bold. Substitutions are located upstream of the LCT gene. The numbers show the distance in nucleotides between the variant and the beginning of the LCT gene (marked as 0).

The identified single nucleotide substitutions and their allele frequencies are shown in Table 1. We found a total of six variants in the region covering 400 bp on both sides of the original C/T_-13910 _variant. The distances between the variants are shown in Figure 1.

**Table 1:** Allele frequencies of single nucleotide substitutions found in this study.

**Origin**	**N**	-13910*T	-13937*A	-14010*C	-14091*T	-14107*A	-14176*C
**Xhosa**	109	0.000	0.014	0.133	0.014	0.005	0.009

**Mixed Ancestry**	62	0.218	0.000	0.065	0.000	0.000	0.000

**Ghana**	196	0.000	0.000	0.000	0.000	0.003	0.000

N = number of subjects

### Xhosa population

The most common substitution in Xhosa population was allele C_-14010 _present in 12.8% of the subjects (Table 1). All the Xhosa subjects were homozygous for the genotype C/C_-13910 _and the T_-13910_-allele was completely absent. In addition, one previously known G/A_-13937 _(rs4988234) and three other novel substitutions C/T_-14091_, G/A_-14107_, A/C_-14176 _were found (Table 1, Figure 1). None of the previously reported variants identified in African and Arab populations (C/G_-13907_, T/C_-13913_, T/G_-13915_) [10-12] were found in this study.

### Mixed ancestry population

The frequency of the T_-13910 _allele was 21.8% and the C_-14010 _allele 8.1% in the mixed ancestry population (Table 1). None of the previously reported mutations (C/G_-13907_, T/C_-13913_, T/G_-13915_, G/A_-13937_) [10,12] or the novel substitutions presented in this study were identified.

### Akan population in Ghana

Both T_-13910 _and C_-14010 _alleles were absent in the Ghanaian population. Only one of the novel substitutions found in the Xhosa population, (G/A_-14107_) in a heterozygous form, was present in (the non-malaria group from) the Ashanti Region in Ghana. None of the other novel substitutions or the previously reported mutations (C/G_-13907_, T/C_-13913_, T/G_-13915_, G/A_-13937_) [10,12] were present in this study population.

## Discussion

In Europe the C/T_-13910 _variant determinates lactase activity in lactase persistence/non-persistence. In pastoralist populations of Africa several other SNPs in the same region upstream of LCT have been identified. Functional evidence for lactase persistence has been obtained for some of these variants [10,12]. We analyzed the distribution of substitutions near C/T_-13910 _in a South African population that consumes milk products and compared it to substitutions found in the Akan peoples from Ghana, who does not have a dairy culture.

Modern humans have lived in South Africa for at least 77,000 years [13]. The early settlers of South Africa, the KhoiKhoi and San people (collectively called the Khoisan), were stone-age hunter-gatherers. They are often thought to represent distinct ethnic groups [14]. Bantu speaking people started to migrate south 2000 years ago bringing with them smelting technology (the iron-age) and expertise in farming [14-16]. Among the Bantu people who migrated to Southern Africa were the Nguni who included the Xhosas, Zulus and Ndebeles. The Xhosa and Zulu people comprise a large proportion of modern day Black South Africans. Xhosa speaking people include many subgroups with related but distinct ancestries [17].

Most of the Xhosa-speaking peoples in today's South Africa, originally came from the Eastern Cape province. They consume fermented milk from the many cattle they keep. This is generally true for most of the Bantu-speaking populations and to some extent indicates their ability to tolerate lactose. We did not find the C/T_-13910 _variant common in populations of European origin, further confirming previously reported findings that this SNP is rare in African populations [10,12,18]. The results of our study show that in the Xhosa population the C_-14010_-allele of the polymorphic site G/C_-14010 _is the most common of those alleles previously associated with lactase activity. The C_-14010 _allele has also been found to be present in Tanzania with a higher prevalence (31.9%) than in the South African Xhosa population [10]. Because of the frequency difference and the fact that the Xhosa population originated from Central Africa and migrated slowly southwards, we hypothesize that the lactase persistence allele has migrated with them and gradually diluted the frequency of C_-14010 _by intermarriages with the Khoisan people [15,16,19].

Interestingly, this study revealed three novel substitutions C/T_-14091_, G/A_-14107_, A/C_-14176 _in the Xhosa population. The substitutions are not reported in the SNP-database . The frequencies of the substitutions are low and unlike the previously detected T/G_-13915_, T/C_-13913 _and C/G_-13907 _they are not located close to the C/T_-13910 _variant in the Oct-1 transcription factor binding site. However, the size of enhancer sites can be in excess of several hundred base-pairs and it is therefore possible that all these variants are included in the same enhancer region [20]. In order to investigate their functional role in the regulation of lactase activity, subjects carrying these mutations need to be recruited and to investigate any associations between them and the results of their lactose tolerance test (LTT) or preferably the lactase activity of their intestinal biopsy specimens. It is known that when the Xhosa people migrated south, they assimilated with the earlier inhabitants of Southern Africa, the Khoisan, thus, it would be interesting to investigate the distribution of these novel substitutions in these populations. However, as they are rare, a large number of samples would be required in order to uncover any functional significance for the three novel substitutions from association studies. Another possibility is that these variants are not associated with lactase persistence but represent the natural diversity of African populations.

Among the people of mixed ancestry origin both the T_-13910 _and the C_-14010 _allele were found reflecting the genetic influence of both Africans and Europeans in this group. This is explained by the diverse ethnic origin (Africans, Western Europeans, the Khoisan, Indonesians and Malaysians) of these people. Therefore the results of the mixed ancestry population group support their ethnic background.

The Akan in the rural Ashanti region live mainly on subsistence agriculture and rarely keep milk producing livestock. Their consumption of fresh milk products has thus remained very limited. This explains why there has not been any evolutionary pressure towards an increasing frequency of lactose persistence genotypes, although these genotypes are known to be present in the population.

## Conclusion

Identification of the G/C_-14010 _variant in the Xhosa population, further confirms their genetic relatedness to other nomadic populations members that belong to the Bantu linguistic group in Tanzania and Kenya. Further studies are needed to confirm the possible relationship of the novel substitutions to the lactase persistence trait.

## Methods

### Study population

The study material consisted of 171 samples collected from Southern Africa for the study of genetic risk factors affecting esophageal cancer [21]. Of these 109 were Xhosa and 62 of mixed ancestry. The Black Xhosa-speaking South Africans originated from the Eastern or Western Capes. The mixed ancestry (commonly referred to as "coloured") subjects are the result of intermarriages between races including Black Africans, Western Europeans, the Khoisan, Indonesians and Malaysians who settled in the Cape from the middle of the 17^th ^century. Written consent was obtained before subjects were enrolled in the study. A questionnaire gathering details on demographics (age, sex, and race), smoking habits and alcohol consumption was completed for all subjects by a trained phlebotomist. Blood was collected from participating subjects and processed for DNA isolation [21]. The study was approved by Cape Town University's Human Research Ethics Committee.

A total of 196 subjects were analyzed from Ghana. Diagnostic procedures and the malariological indices of the women have been described elsewhere [22]. DNA was extracted from the subjects' peripheral venous blood (QIAmp, Qiagen). All the participants gave their informed consent. The study protocol was reviewed and approved by the Committee on Human Research Publication and Ethics, School of Medical Sciences, University for Science and Technology, Kumasi, Ghana, and the institution's guidelines were followed in conducting the study.

### Genotyping

The DNA fragment spanning the C/T_-13910 _variant was amplified by polymerase chain reaction (PCR) and then sequenced. The primer sequences used were: 5'-CCTCGTTAATACCCACTGACCTA-3' (forward) and 5'-GTCACTTTGATATGATGAGAGCA-3' (reverse). The PCR products were separated on a 1.5% agarose gel containing ethidium bromide for size verification. The excess primers were removed by digestion with 2.5 U of Shrimp Alkaline Phosphatase (USB) and 5 U of Exonuclease I (New England Biolabs) at 37°C for 45 min followed by inactivation at 80°C for 15 min. For sequencing the BigDye 3.1 terminator (Applied Biosystems) was used according to the manufacturer's instructions. The sequencing reaction was as follows: at 96°C for 1 min, then 25 cycles at 96°C for 10 s, at 55°C for 5 s and at 60°C for 4 min, which was followed by purification with Millipore Multiscreen plates (Millipore, USA) with Sephadex G-50 Superfine sepharose (Amersham Biosciences, Sweden), electrophoresis with an ABI 3730 DNA Analyzer (Applied Biosystems) and base calling using the Sequence Analysis 5.2 software (Applied Biosystems). The results were analyzed by Sequencher 4.1.4 software (Gene Codes, USA).

## Authors' contributions

ST carried out sequencing, analysed sequences, drafted the manuscript and made the figure and the table. CD and IP provided the samples from South Africa and their expertise in drafting the manuscript. VH provided samples from Ghana and description of this study population and participated in drafting of the manuscript. EL participated in the laboratory work and drafting. IJ got the idea for the study, participated on designing of the study and final drafting of the manuscript. All authors read and approved the final manuscript.
